# Flower-Like ZnO Nanorods Synthesized by Microwave-Assisted One-Pot Method for Detecting Reducing Gases: Structural Properties and Sensing Reversibility

**DOI:** 10.3389/fchem.2020.00456

**Published:** 2020-07-02

**Authors:** Abdullah Aljaafari, Faheem Ahmed, Chawki Awada, Nagih M. Shaalan

**Affiliations:** ^1^Department of Physics, College of Science, King Faisal University, Al-Ahsa, Saudi Arabia; ^2^Physics Department, Faculty of Science, Assiut University, Assiut, Egypt

**Keywords:** nanoflowers, ZnO nanorods, gas sensor, methane, carbon monoxide, hydrogen

## Abstract

In this work, flower-like ZnO nanorods (NRs) were successfully prepared using microwave-assisted techniques at a low temperature. The synthesized NRs exhibited a smooth surface and good crystal structure phase of ZnO. The sharp peak of the XRD and Raman spectrum confirmed the high crystallinity of these ZnO NRs with a pure wurtzite structure. The nanorods were ~2 μm in length and ~150 nm in diameter, respectively. The electron diffraction pattern confirmed that the single crystal ZnO nanorods aligned along the [001] plane. The NRs were applied to fabricate a gas sensor for reducing gases such as CH_4_, CO, and H_2_. The sensor showed a good performance and sensitivity toward the target gases. However, its response toward CH_4_ and CO was higher compared to H_2_ gas. Although the operating temperature was varied from room temperature (RT) up to 350°C, the sensor did not show a response toward any of the target gases in the range of RT-150°C, but dramatic enhancement of the sensor response was observed at 200°C, and up to higher temperatures. This behavior was ascribed to the activity of the smooth surface and the reactivity of surface oxygen species with the targeted gases. The sensor response was measured at various gas concentrations, where the calibration curve was shown. The gas sensing mechanism was described in terms of the reaction of the gases with the transformed oxygen species on the surface of the oxides.

## Introduction

Due to their optical and physical properties, ZnO nanostructures have become a suitable material to use in different environmental monitoring applications (Livage, 1981; Roy et al., 2011; Spencer, 2012; Brookes et al., 2014; Kumar et al., 2015; Chen et al., 2019; Zada et al., 2019, 2020; Qi et al., 2020a,b). One of these applications that have attracted the interests of scientific communities is gas–sensing applications that require some specific characteristics such as high surface-to-volume ratio and good chemical and thermal stability (Vomiero et al., 2007; Guo et al., 2012; Pan et al., 2013; Bai et al., 2014; Hosseini et al., 2015; Jin et al., 2015; Mascini et al., 2018; Jingxuan et al., 2020).

In fact, the morphology of materials-based gas sensors is extremely important to the performance of the gas sensor. Thus, since the first use of ZnO nanostructures as gas-sensing materials (Seiyama et al., 1962), many studies have been reported on the morphology-dependent gas sensor performance (Alam et al., 2015; Zhang et al., 2017; Ridha et al., 2018; Qin et al., 2019; Zhao et al., 2019). Zhang et al. showed that 1DZnO nano-cones were highly promising for practical application in gas sensors, due to their large surface area per unit mass and unique conical structure (Zhang et al., 2017). Uniform 1D ZnO/ZnCo_2_O_4_ nanocomposite showed a fast response and good selectivity to acetone gas (Qin et al., 2019). In the same sequence, an intensive investigation on the effects of the lengths and diameters of ZnO nanorods, with regard to the sensing performance of ethanol vapor, was also studied (Ridha et al., 2018). ZnO NRs supported by the complex surfactant showed excellent ethanol sensing properties at an optimal operating temperature of 300 °C, which could be attributed to their large surface to volume ratio, and a high number of surface defects due to oxygen vacancies (Zhao et al., 2019). A new structure, by synthesizing Pd nanocubes (NCs) decorated on vertical ZnO nanorods (NRs) applied to a resistive-type gas sensor, was developed by Bin Alam et al. (2015). The shape control of Pd NCs with close {111} packing effects remarkably enhances the catalytic activity and capacity for H_2_ adsorption compared to that of {100}.

The challenges are not limited to the high-performance gas sensor but also to the synthesis methods, as few of them consider the real working conditions of a sensor during the functional characterizations, especially, when they address complex structures. For example, the electrochemical method (Lee and Tak, 2001), template-based growth (Li et al., 2000), sol-gel processing (Chen and Liu, 2005), and the hydrothermal method (Rakshit et al., 2012) are some of the techniques widely used for growing different morphologies of ZnO nanostructures. More specifically, many methods have the disadvantages of low productivity, long processing times, and low growth rates. Time is invaluable and becoming increasingly important in these manufacturing processes where one has to advance in many trial and error experiments to obtain the best results. In this regard, microwave synthesis methods have unique influences on high reaction rates, short processing times, reaction selectivity, power-saving, and are low cost (Rana et al., 2016).

It is known that gas sensing properties strongly depend on the morphology of the sensing materials. Various ZnO nanostructures, such as nanoparticles, quantum dots, cloudy-like nanoparticles, isotropic nanoparticles, and nanorods were investigated as a sensitive layer for various gasses, and it was found that the morphology of ZnO nanostructures significantly influenced the responses of the sensors to the reducing gases (Joncaa et al., 2014; Park et al., 2019). ZnO nanostructures of one-dimension such as rods, wires, tubes, and belts have been attracting increased attention due to their aspect ratio, high surface to volume ratio, high electron mobility, etc.; these parameters play an important role in the gas sensor (Hernandez-Ramirez et al., 2009). While working with the 1D structure, we might observe a considerable diversity in geometric parameters of this nanostructure. Thus, if the 1D nanostructure has been used as a sensitive layer, specific geometrical parameters are considered. Shaalan et al. (2011) found that the sensing properties of oxide gas sensors were well-controlled by the 1D nanostructure, and although the high surface-to-volume ratio of the sensing layer was an important parameter to enhance the sensing response, the low density of the potential barrier at interconnected grains was required and had better be considered in the proposal of sensors. The results expressed that the 1D structure has many advantages in reliability and high response.

It was reported that the flower-like structure of random branches was helpful for avoiding agglomeration as well as showed good electron transportation. This structure has been reported for ZnO with high performance in photocatalysts (Bohle and Spina, 2009), chemical sensors (Wan et al., 2004), etc. The high performance is ascribed to the surface defects, species, and surface adsorption. Wang et al. developed flower-like ZnO on GaN using the electro-deposition technique and its application as ethanol gas sensors at room temperature (Wang et al., 2019). Fan et al. combined the hydrothermal method with electrospinning to produce flower-like ZnO hierarchical structures that showed high sensitivity toward H_2_S gas (Fan et al., 2019). Agarwal et al. showed that nanorods-like ZnO structures synthesized using the hydrothermal method were very selective and sensitive toward NO_2_, but not CO (Agarwal et al., 2019). Although the improvement carried-out on the gas sensor performance due to the morphologies effects presented in the previous studies, the morphology and structure geometry in gas-sensing properties for developing reliable and sensitive gas sensors are still highly considered.

In this work, flower-like ZnO nanorods (NRs) are fabricated in a microwave oven. Low growth temperature and a short time are used in the current work for growing polycrystalline ZnO nanorods. The procedures used in this method are simpler than the traditional method. The structure and morphology of the synthesized ZnO NRs were studied by XRD, FE-SEM, TEM, and Raman spectroscopy. These NRS are applied in fabricating gas sensors for testing its sensitivity in detecting different reducing gases at different operating temperatures. The gas sensor was applied to investigate the reversible behavior toward CH_4_, CO, and H_2_ reducing gases with repeated cycles and various gas concentrations. Carbon monoxide (CO) gas is generally regarded as one of the most dangerous air pollutants among greenhouse gases and is produced from exhausts of factories, and vehicles. It is odorless, colorless, and regarded as a silent killer gas. CH_4_ is highly combustible and can form an explosive mixture with ambient air. Thus, it is important to monitor CH_4_ escaping into the atmosphere for both industrial process control and reduction of environmental pollution. Hydrogen gas is used as a potential fuel in vehicles and fuel cells to be transformed into electricity. It is also used in the production of some industrial chemicals and food products. An explosion can occur if hydrogen leaks into the air at a specific level (4%). Therefore, there is a need to develop a reliable sensor based on metal oxide reducing gas with improved performance. Our work demonstrates the developing possibility of a ZnO-nanostructure based gas sensor for reducing gases.

## Materials and Methods

### Preparation and Characterization of ZnO Nanorods

All reagents used in this research were of analytical grade and used as received. The synthesis was performed in a simple microwave oven (Samsung, 750 W). For the synthesis of nanorods, a reaction solution in 100 ml deionized water was prepared to contain a 1:20 molar ratio of zinc acetate dihydrate [Zn(CH_3_COO)_2.2_H_2_O; 99.99%, Sigma Aldrich] and potassium hydroxide (KOH; 99.99%, Sigma Aldrich), and then transferred into a commercial microwave oven. The reaction was carried out at a microwave power of 180 W for 20 min (Ahmed et al., 2011). Subsequent to microwave reaction, the resulted solution was cooled to room temperature and the precipitate was obtained. The resulted precipitate was separated by centrifugation, followed by thorough washing in the presence of deionized water and ethanol numerous times, and the collected samples were finally dried in an oven at 80°C for 24 h. The sample was calcined at 400°C for 2 h and then used for the gas sensing measurements.

**Figure 1:**
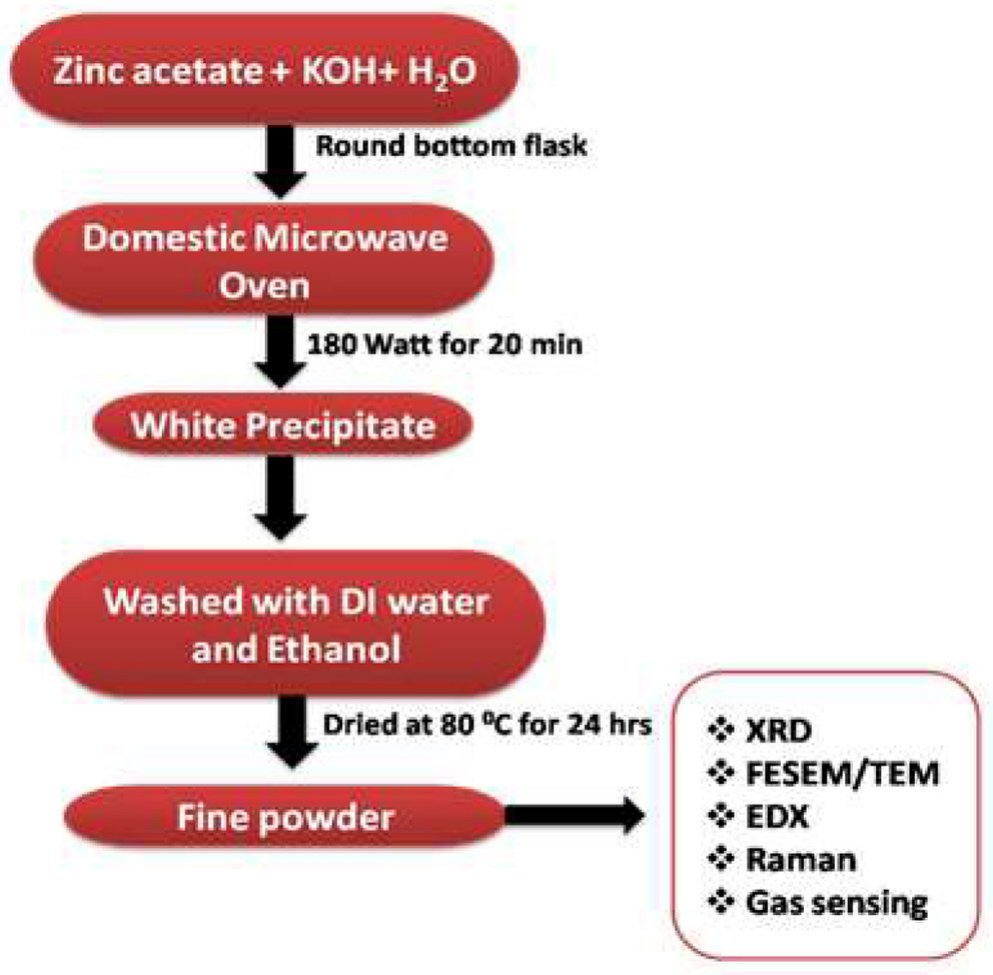
Flowchart scheme diagram of the synthesis procedures of ZnO NRs.

X-ray diffraction [Phillips X'pert (MPD 3040)] was used to study the crystal structure and phase purity of the samples. Morphologies of the samples were studied by Field emission scanning electron microscopy (FESEM) (TESCAN; MIRA II LMH microscope). To find the elemental composition of ZnO, energy dispersive X-ray spectroscopy (EDX, Inca Oxford, attached to the FESEM) was used. Further morphological characterization including micrographs, selected area electron diffraction (SAED) pattern, and high-resolution transmission electron microscopy (HRTEM) of the samples were performed by Transmission electron microscopy (TEM) [FE-TEM (JEOL/JEM-2100F version] operated at 200 kV. The Raman spectra were measured using a LabRAM HR800 confocal micro-Raman spectrometer equipped with a multichannel charge-coupled detector. A He-Cd laser (wavelength 442 nm, 20 mW) was used as a source of excitation. The number of gratings in the Raman spectrometer was 1,800 l/mm. The Raman spectra were collected in a backscattering geometry with a spectral resolution of 0.8 cm^−1^ at ambient temperature. Figure 1 shows the flow diagram for the synthesis of ZnO nanostructures.

### Preparation and Characterizations of Gas Sensor

**Figure 2:**
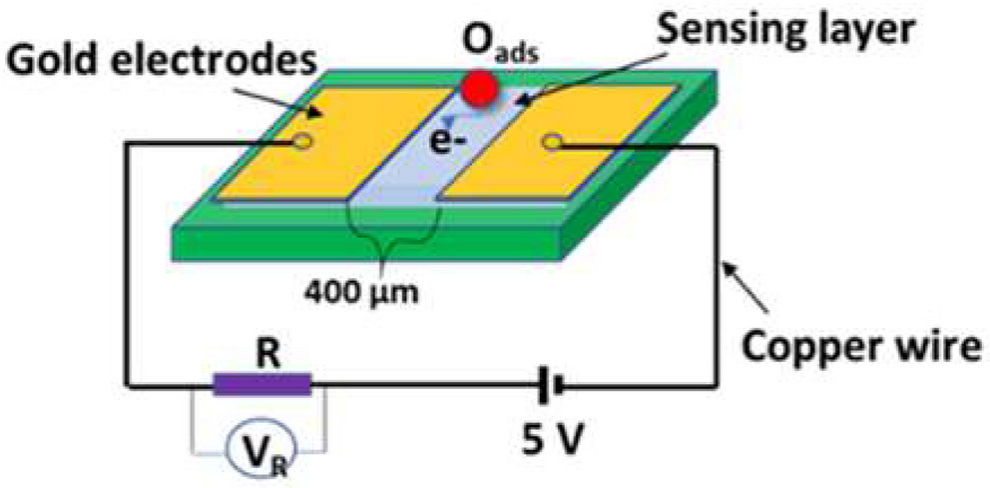
Schematic diagram of the device and electrical connections.

Since the sensing materials are in powder form, the sensor fabrication was carried out in a sequence of two gold electrodes with 400 μm-gap deposited by DC-sputtering on a substrate of glass. The sensing layer of 10 μm was deposited by the screen-printing method on the top of the electrodes, as shown in Figure 2. The sensing layer was then treated for 30 min at 400°C in the air ambient before testing the target gases in order to avoid any recrystallization during the testing. The operating temperature starting from 200 up to 350°C was well-controlled. Dry synthetic air (21%O_2_, and 79% N_2_) mixed with a gas such as H_2_, CO, and CH_4_ flowed into the chamber at a flow rate of 200 SCCM, which was controlled by Horiba MFCs (SEC-N112 MGM-Horiba). A computerized data acquisition instrument (Multi-channel- LXI-Agilent 34972A) was used to record the electrical measurements. The sensing response calculated form the electrical data is defined as *S* = R_a_/R_g_, where R_a_ and Rg are the sensor resistances in the air and gas, respectively.

## Results and Discussions

### Structure and Morphology

**Figure 3:**
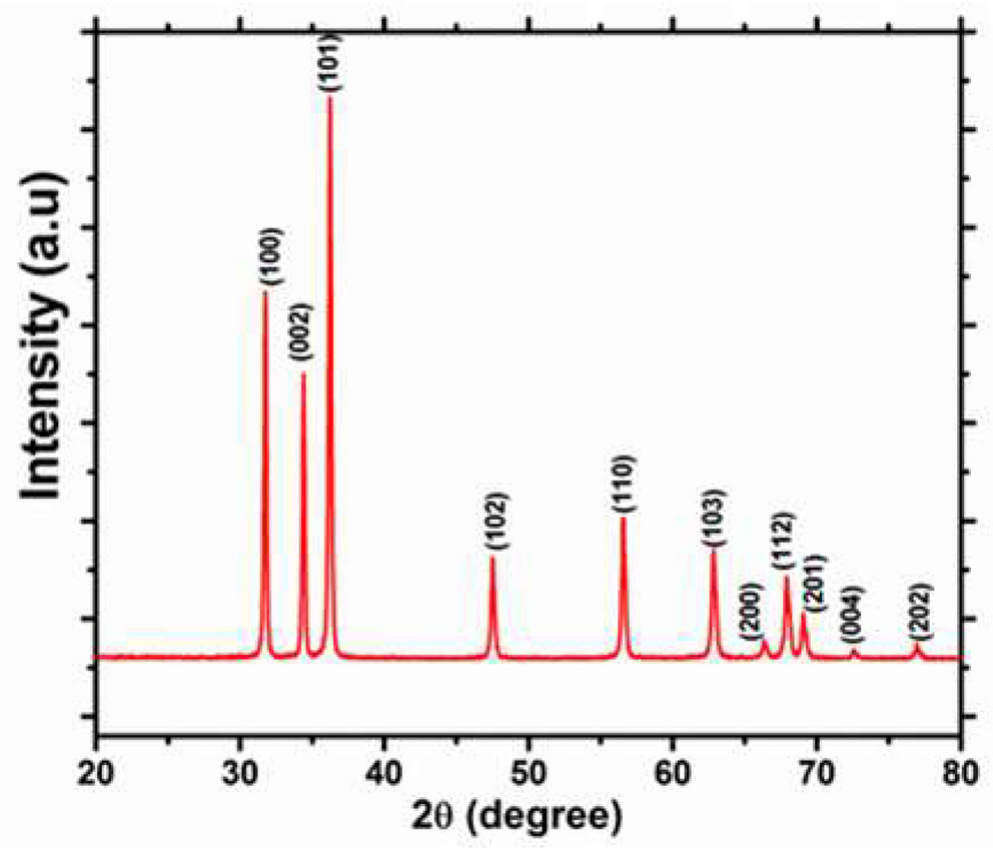
XRD patterns of ZnO nanorods.

The XRD pattern of the as-prepared ZnO powder is shown in Figure 3. The chart is indexed in the hexagonal phase with the lattice-matched parameters (a = 3.254 Å and c = 5.197 Å), which are very close to the standard data reported in the JCPDS, 89-0501 card. The recorded XRD pattern exhibits a single-phase nature for the wurtzite-ZnO structure. The XRD pattern does not show any diffraction peaks for other impurities, and the high crystallinity is shown from the sharpness of the peaks of the as-prepared ZnO nanorods.

**Figure 4:**
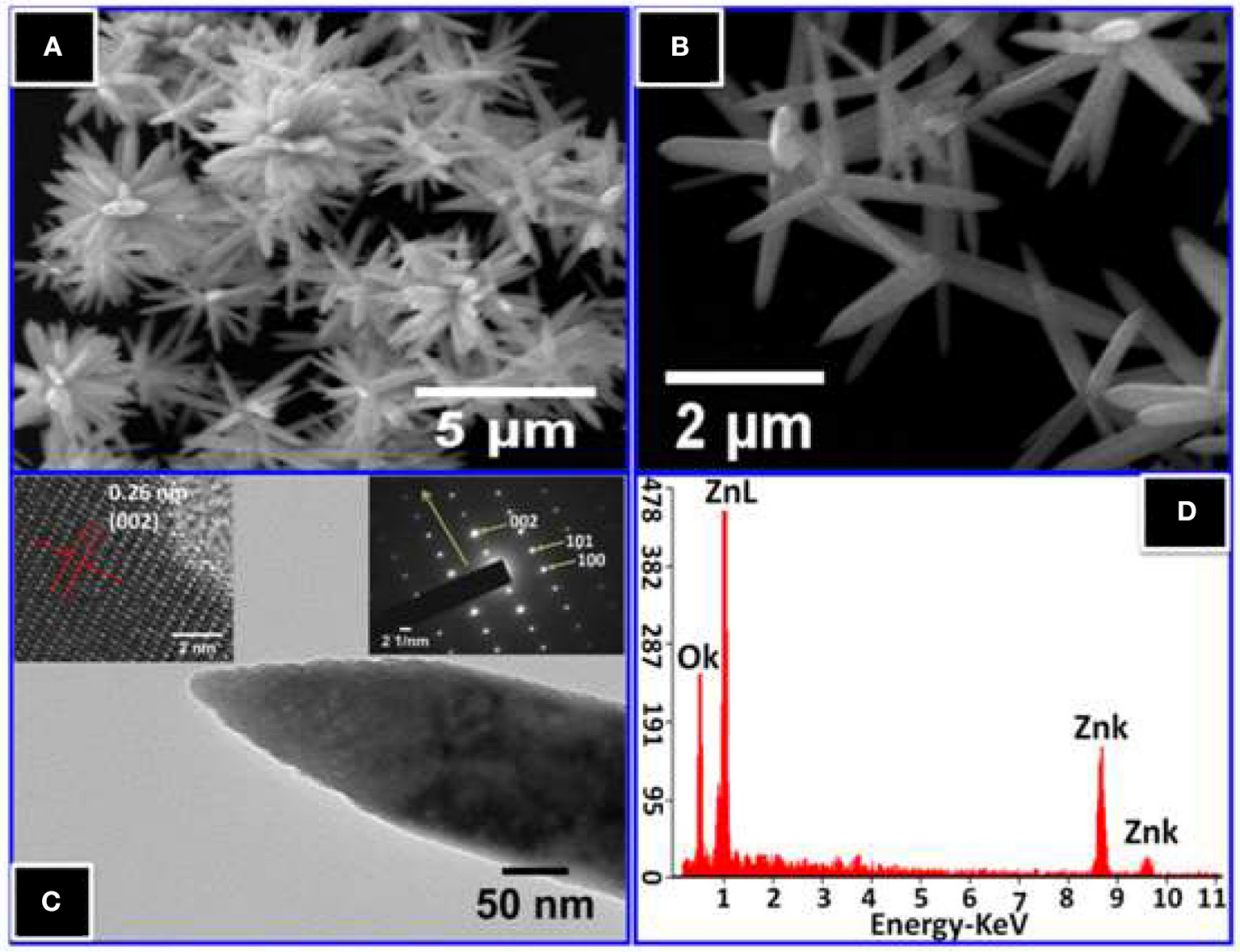
**(A)** Low magnification and **(B)** high magnification FESEM images of ZnO nanorods. **(C)** The TEM image of a single ZnO nanorod, the upper left inset shows HRTEM and right insets show SAED pattern of a single nanorod, **(D)** EDS spectrum of ZnO nanorods.

Figures 4A,B show FESEM images of ZnO nanostructures. The images exhibit flower-like clusters for the synthesized ZnO nanorods on a large-scale with high dispersion, and more or less uniform morphologies. From the high magnification image shown in Figure 4B of flower-like ZnO, numerous symmetric taper arms composed of a number of aggregative nanorods can be observed.

Figure 4C shows a complementary morphological description obtained by TEM with SAED. From this figure, the TEM micrograph of a typical individual ZnO nanorod confirms the crystal quality and growth direction. The diameters of the NRs range from 145 to 185 nm with a tip diameter of ~15 nm, while the length is approximately 2 μm. Further, the TEM image clarifies, that the ZnO nanorod has a sharp tip at the end. In addition, HRTEM shows a well-resolved d-spacing of 0.265 nm, which corresponds to the plane (002) of wurtzite ZnO, indicating single crystallinity for the ZnO NRs in nature with and preferentially growth direction of [001] in c-axis, which was confirmed in SAED pattern. For further confirmation, the EDS spectrum of the elemental analysis of ZnO NRs is shown in Figure 4D. The only Zinc and oxygen signals are detected in the spectrum, which confirms that the NRs are pure ZnO.

A LabRAM HR800 confocal micro-Raman spectrometer is used to measure the Raman spectra. As one of the simplest uniaxial crystals, ZnO with a wurtzite structure belongs to the ${\text{C}}_{6\text{v}}^{4}$ (P6_3_ mc) space group. For the perfect ZnO crystal, only the optical phonons at Γ point of the Brillouin zone are involved in first-order Raman scattering. We can see different optical modes in the group theory: Γopt = A_1_+2B_1_+E_1_+2E_2_. The two first modes, A_1_ and E_1_, are polar and can be divided into transverse optical (${\text{A}}_{1}^{\text{TO}}$ and ${\text{E}}_{1}^{\text{TO}}$) and longitudinal optical (${\text{A}}_{1}^{\text{LO}}$ and ${\text{E}}_{1}^{\text{LO}}$) components. E_2_ mode is composed of two modes, a low and a high-frequency phonon (${\text{E}}_{2}^{\text{low}}$ and ${\text{E}}_{2}^{\text{high}}$), which correspond to the vibration of the heavy Zn sublattice and oxygen atoms, respectively. According to the Raman selection rule, the modes mentioned above are first-order Raman-active modes (Zhang et al., 2005, 2009). For the B_1_ mode, it is silent and has two frequencies which are the ${\text{B}}_{1}^{\text{low}}$ and ${\text{B}}_{1}^{\text{high}}$ modes, located at 260 and 540 cm^−1^, respectively (Damen et al., 1966; Calleja and Cardona, 1977).

**Figure 5:**
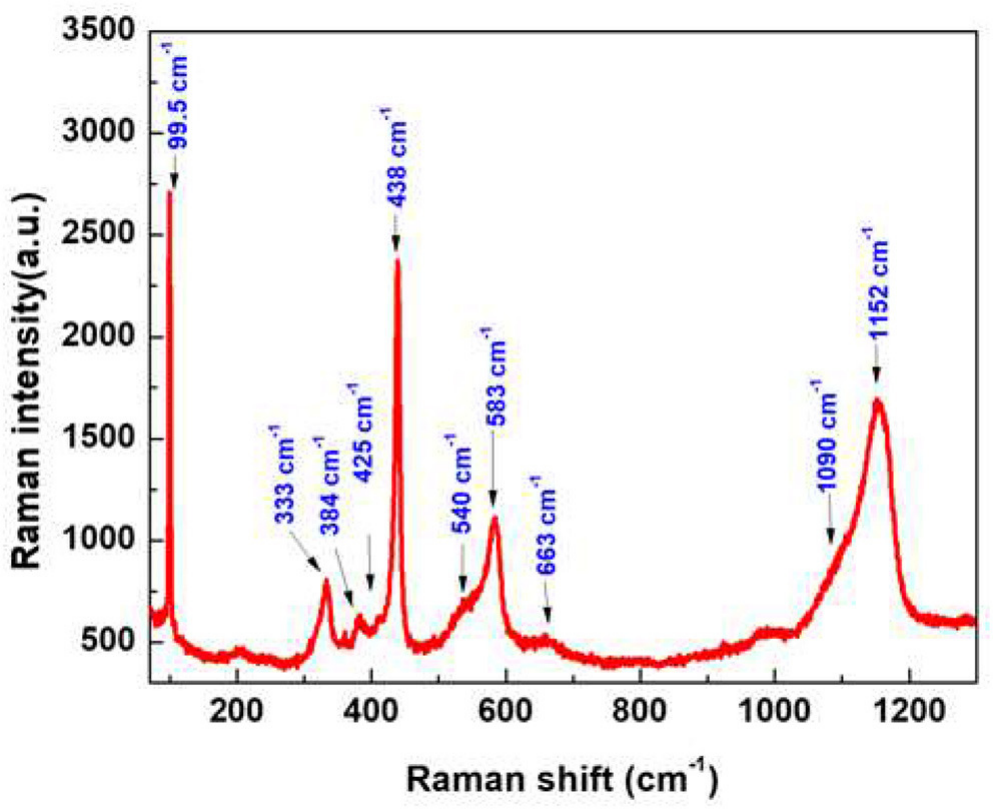
Room temperature Raman spectrum of ZnO nanorods.

In our geometry of excitation and collection, the E_2_, A_1_ (TO), and E_1_ (TO) modes are active when the incident light is perpendicular to the c-axis (Decremps et al., 2002). As the c-axis is oriented in space, most of the modes can appear. For example, the ${\text{E}}_{2}^{\text{low}}$ mode is observed at 99.5 cm^−1^ and the ${\text{E}}_{2}^{\text{high}}$ mode at 438 cm^−1^ has a high intensity in the Raman spectrum (Figure 5), confirming perfect crystallinity of the sample. The peak located at 384 cm^−1^ is assigned to the A_1_ (TO) mode. By performing the Gaussian–Lorenz fitting, we can observe a weak shoulder peak located at 425 cm^−1^ that corresponds to the E_1_(TO) mode. The E_1_(LO) mode is observed at 583 cm^−1^; this peak can be observed with the c-axis of nanorods which is normal on the surface of the sample. Moreover, we can also observe another optical phonon mode near the A_1_ symmetry, located at 333 cm^−1^ (Rajalakshmi et al., 2000). The acoustic combination of A_1_ and E_2_ was observed around 1,101 cm^−1^ (Wang et al., 2004). Our results show a large band located between 1,060 and 1,200 cm^−1^, which are in good agreement with previous literature's report. The broad peak at 663 and 1,152 cm^−1^ are due to the multi-phonon process (Calleja and Cardona, 1977). We can also clearly see one of the silent modes, B_1_ (high), which is located at 540 cm^−1^.

### Sensing Properties

**Figure 6:**
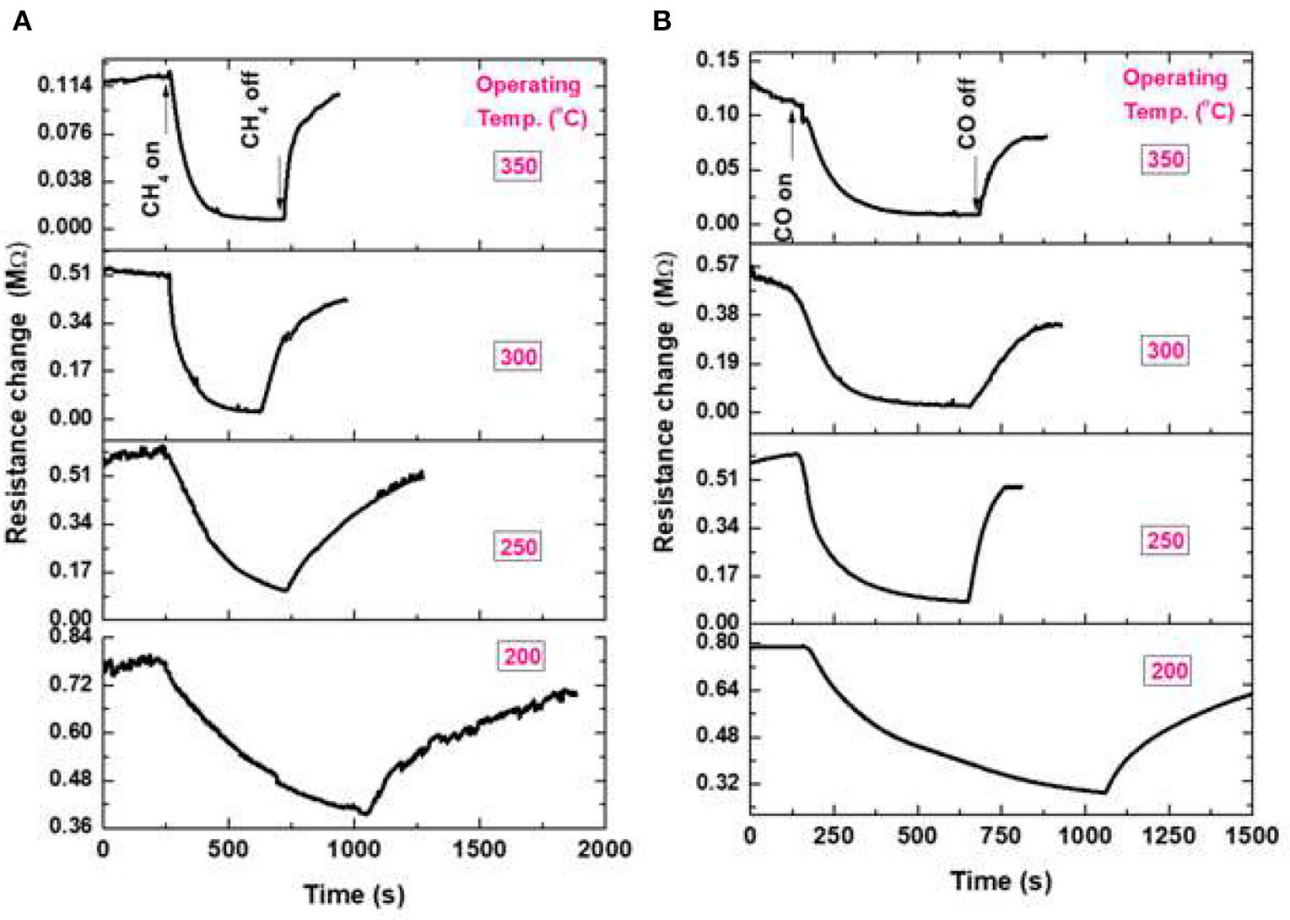
Sensor signal of ZnO NRs at various operating temperatures; **(A)** toward 1% CH_4_, and **(B)** toward 0.1% CO.

The sensing properties of ZnO flower-like NRs sensors are systematically studied at various operating temperatures toward reducing gases of CH_4_, CO, and H_2_. The variation of resistance in the presence of gases for the fabricated NRs sensor toward 1.0% CH_4_, 0.1% CO, and 1.0% H_2_ is shown in Figure 6. It can be seen that the resistance of the sensor decreases if the gas is introduced to the sensor surface, suggesting that the prepared ZnO nanostructure is an n-type semiconductor-like material. According to the band theory (Yamazoe et al., 1979) in gas sensors, the target gas interacts with the surface of the metal oxide semiconductor through surface adsorbed oxygen ions. The interaction causes a change in the charge carrier concentrations of the oxide, resulting in a resistivity change. The majority of carriers of n-type semiconductors, such as ZnO, are electrons. Upon exposure to the reducing gas, a decrease in resistivity occurs because the electrons are injected back to the conduction band of the oxide. This explanation is in good agreement with the presented results in this work. The operating temperature of the sensor is varied from RT up to 350°C, however, the sensor did not show any response in the range of RT-150°C (not shown here). On the contrary, a dramatic change in the sensor response is observed at 200°C, which is ascribed to the increase of the surface activity of NRs at higher temperatures, since the gas reacts with the active sites on the oxide surface. Although ZnO NRs have a high surface area compared to the bulk, the surface of ZnO NRs presented here is smooth, which has a low surface activity to the gas reaction at the low temperature.

To explain the surface activity, we must clarify the reaction of surface species (adsorbed molecules) in air and in air containing gas. It is well-known that ZnO is an n-type semiconductor, and its gas-sensing mechanism belongs to the surface-controlled type (Ruhland et al., 1998; Koziej et al., 2007; Haridas and Gupta, 2012; Shaalan et al., 2019a,b), and the change in conductivity is dependent on the species type and the amount of chemisorbed oxygen on the surface. The intrinsic resistance of the semiconductor decreases when increasing the temperature; however, we have to consider the transformation of physisorbed oxygen molecules into various oxygen ions when increasing the surface temperature. Ruhland et al. (1998), have supposed the transformation of oxygen molecules with surface temperature as shown in the following equations:

(1) $$\begin{array}{l} O_{2}\left(g\right)+e^{-}\to O_{2}^{-},\left(\text{below }200{}^{\circ}\text{C}\right) \end{array}$$

(2) $$\begin{array}{l} O_{2}^{-}+e^{-\;}\to 2O^{-},\left(\text{above }200{}^{\circ}\text{C}\right) \end{array}$$

(3) $$\begin{array}{l} O^{-}+e^{-}\to O^{2-},\left(\text{above }300{}^{\circ}\text{C}\right) \end{array}$$

**Figure 7:**
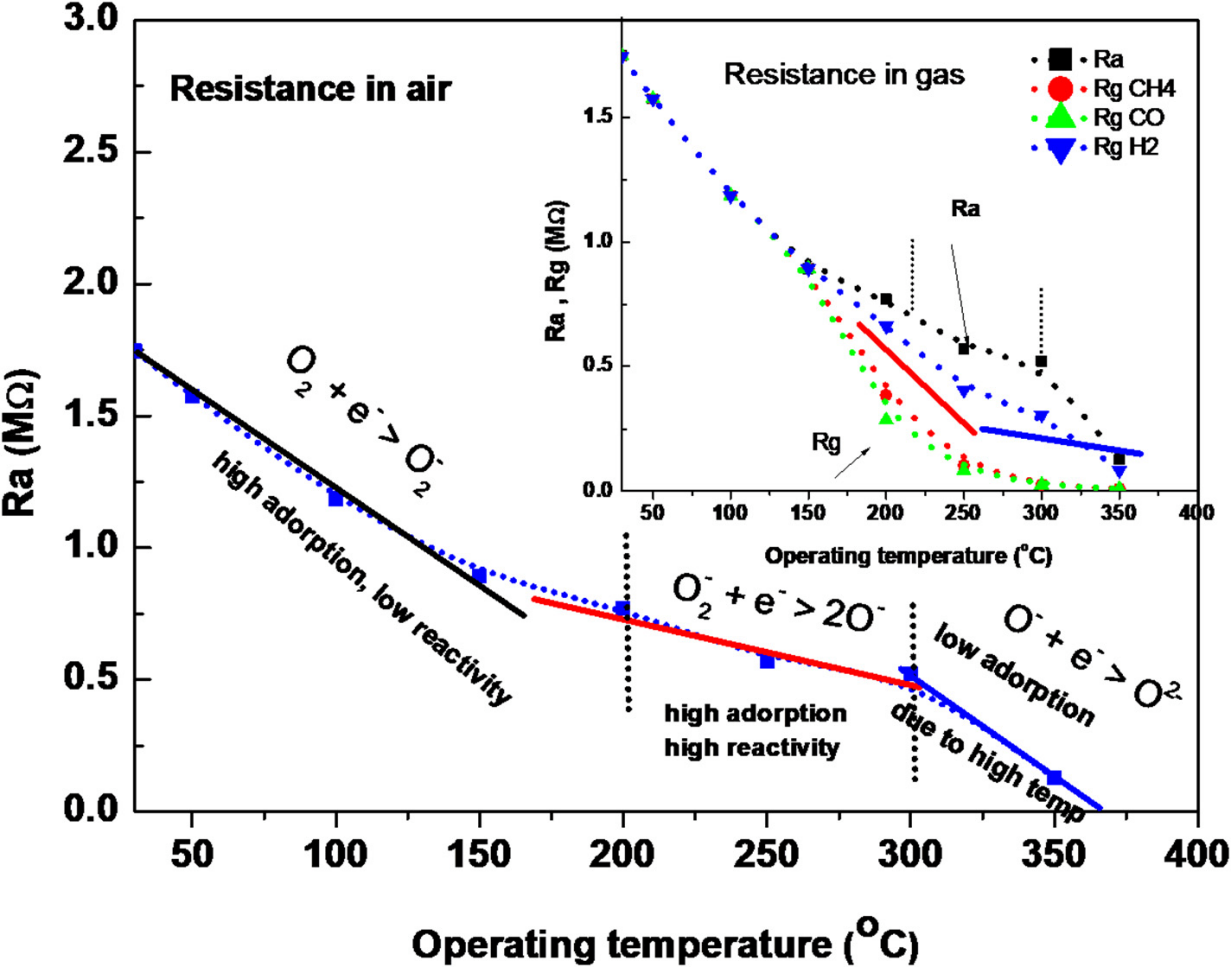
The change in resistance in air and in air having gas as a function of temperature.

Thus, studying the behavior of oxide in the air with these transformations compared to the intrinsic behavior of the semiconductor, we may be able to understand the surface activity at various temperatures. Figure 7 shows the change in the ZnO conductivity (based resistance change) in air and in gas (inset figure) as a function of the operating temperature. It indicates that the charge exchange reactions of ZnO NRs with the oxygen species are dependent on the surface temperature. Three regions can clearly distinguish the reaction of oxygen species with ZnO electronic surface in air. The first region shows that ZnO conductivity increased with an increasing temperature, although O_2_ molecules transform to ${\text{O}}_{2}^{-}$, which picked up some electrons from the conduction band of the oxide. However, the electron transport in the conduction band due to thermal energy, dominates the conduction, indicating a low reactivity of oxygen onto the ZnO surface at these low temperatures of RT-150°C. In this temperature range, the resistance of the ZnO NRs sensor (in the air) decreased with the large slope with an increasing surface temperature. In the second range of 200–300°C, it decreases with a low slope, which is expected to be due to the high reactivity of the dissociation of O^2−^ to 2O^−^ with the surface of ZnO. The third region shows, again, a large decrease in ZnO resistance, although 2O^−^ transforms to O^2−^. This is attributed to the low adsorption and diffusion of oxygen molecules into the grains of ZnO at this high temperature, which allows for more conduction electrons to thermally transport.

It is well-known that reducing gas prefers to react with atomic oxygen ions *O*^−^ rather than $O_{2}^{-}$ ions on the surface, which causes the sensor to be active at 200°C (Ruhland et al., 1998; Koziej et al., 2007; Haridas and Gupta, 2012; Shaalan et al., 2019a,b). Thus, at low temperatures of RT-150°C, the chance of reaction is very low. However, it is highest at 200–300°C when *O*^−^ ions are the most available species. This reactivity decreases again at higher temperatures of 350°C due to the formation of $O_{2}^{-}$. Thus, the dramatic improvement in the response at 200–300°C can be attributed to the light of the formation of oxygen species. As a result of the forming oxygen ions on the oxide, the reducing gas reacts with *O*^−^ ions and produces neutral compounds (such as H_2_O, and CO_2_ depending on the gas type Koziej et al., 2007; Haridas and Gupta, 2012; Shaalan et al., 2019a,b) and injects electron charges back to the oxide conduction band, causing a decrease in the resistance due to the increase in the conduction electron density, as follows:

(4) $$\begin{array}{l} CH_{4}+4O_{\left(ads\right)}^{-}\to CO_{2\left(gas\right)}+2H_{2}O_{\left(gas\right)}+4e^{-}\\ \;\left(\text{complete reaction}\right) \end{array}$$

(5) $$\begin{array}{l} CO+O_{\left(ads\right)}^{-}\to CO_{2\left(gas\right)}+e^{-} \end{array}$$

(6) $$\begin{array}{l} H_{2}+O_{\left(ads\right)}^{-}\to H_{2}O_{\left(gas\right)}+e^{-} \end{array}$$

The sensor response measured for ZnO NRs at various operating temperatures of 200–350°C with exposure to 1% of CH_4_ is shown in Figure 6A. When the temperature increased from 200 up to 350°C, the sensing performance enhanced, as well as the response/recovery times constants. The decrease in resistance upon exposure to CH_4_ was assumed by Haridas and Gupta (Haridas and Gupta, 2012) to be due to the dissociation of methane molecules, which react with the surface O^−^ species, liberating the trapped charges to the oxide, reducing its resistance. The gas sensing signal of ZnO NRs at a temperature of 200–350°C with exposure to 0.1% CO gas is shown in Figure 6B. With the increase in operating temperature, an enhancement in the sensing response behavior is observed. Koziej et al. (2007) have predicted a reaction mechanism for the reducing gas such as CO with oxygen ions on the oxide. Upon the reaction of CO with the *O*^−^ ions, an electron was released to the conduction band of the oxide, increased the oxide conductivity. Reviewing the basic reaction between H_2_ gas and oxide surface is described in terms of the reaction of H_2_ with the oxygen species as the following single-step process (Koziej et al., 2007). In this reaction, H_2_O (gas) is produced as a final product, followed by the accumulation of electron charges at the oxide surface causing the conductance increase (*not shown here*).

### Operating Temperature Control

**Figure 8:**
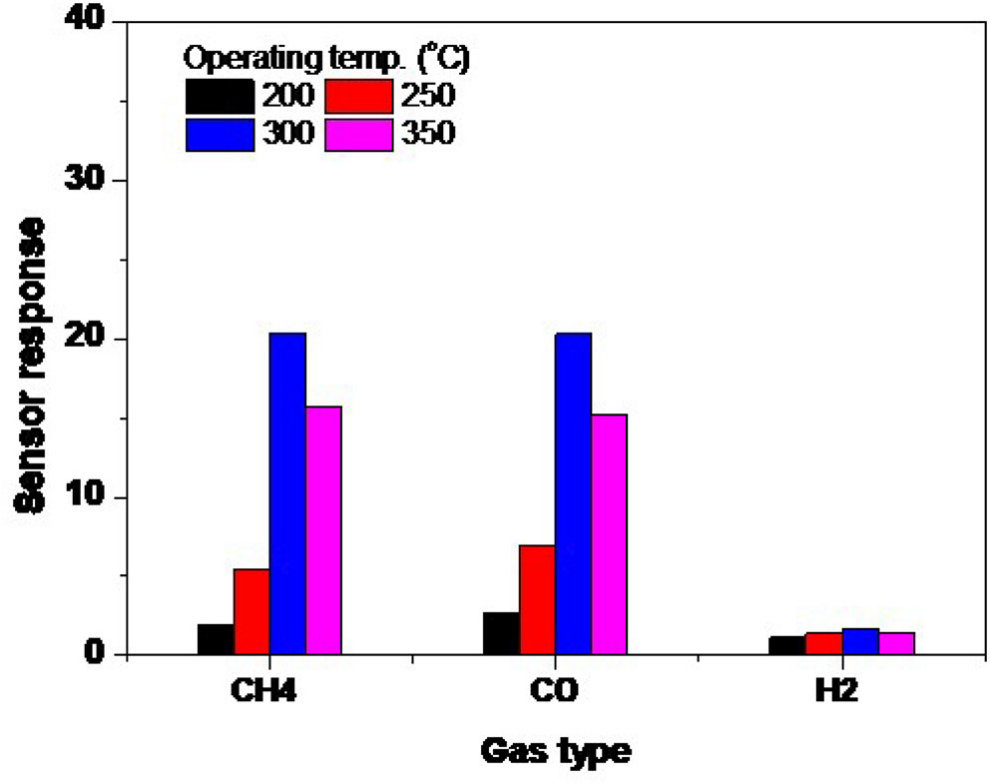
The sensor response variation of NRs sensors with exposure to 1.0% CH_4_, 1.0% H_2_, and 0.1% CO at various operating temperatures.

The dependence of sensing behavior on the operating temperature is a very important parameter to describe the gas sensor, in order to obtain the highest performance of this sensor. Figure 8 shows the response of the fabricated sensor at various operating temperatures, starting from 200 up to 350°C. The response was well-calculated for CH_4_, H_2_, and CO gases. The response of the sensors fabricated from NRs is shown in Figure 8. It seems that the sensing layer is more active when increasing the operating temperature, while in the NRs sample the maximum response occurs at a high temperature of 300°C. When the temperature was increased further, a weaker response was observed. The sensor gains the same behavior for all gases; however, it is less sensitive toward H_2_ gas. The maximum responses are recorded as high as 20.2 for CH_4_ and CO gases, while it is 1.7 for H_2_ gas at 300°C. We may ascribe the low sensitivity of ZnO NRs toward H_2_ to the low reactivity of H_2_ with the smooth surface of the oxide. To react H_2_ with the surface in a better way, the surface catalytic is favorably introduced to assist the dissociation of H_2_ as in the case of the Spillover effect.

The decrease in response at higher temperatures is possible due to the quantity of the diffused oxygen and gas into the oxide. The diffusion and adsorption are exothermic phenomena, so at high temperatures the amount of the diffused species into the surface decreases, which leads to a lower response. In addition, at high temperatures, the *O*^−^ ions mostly transform into *O*^2−^ ions, which is unlikely to react with reducing gases. However, we may consider that *O*^−^ ions are still there with in low amounts, which reduces the sensing response.

### Gas Sensing Evaluation

**Figure 9:**
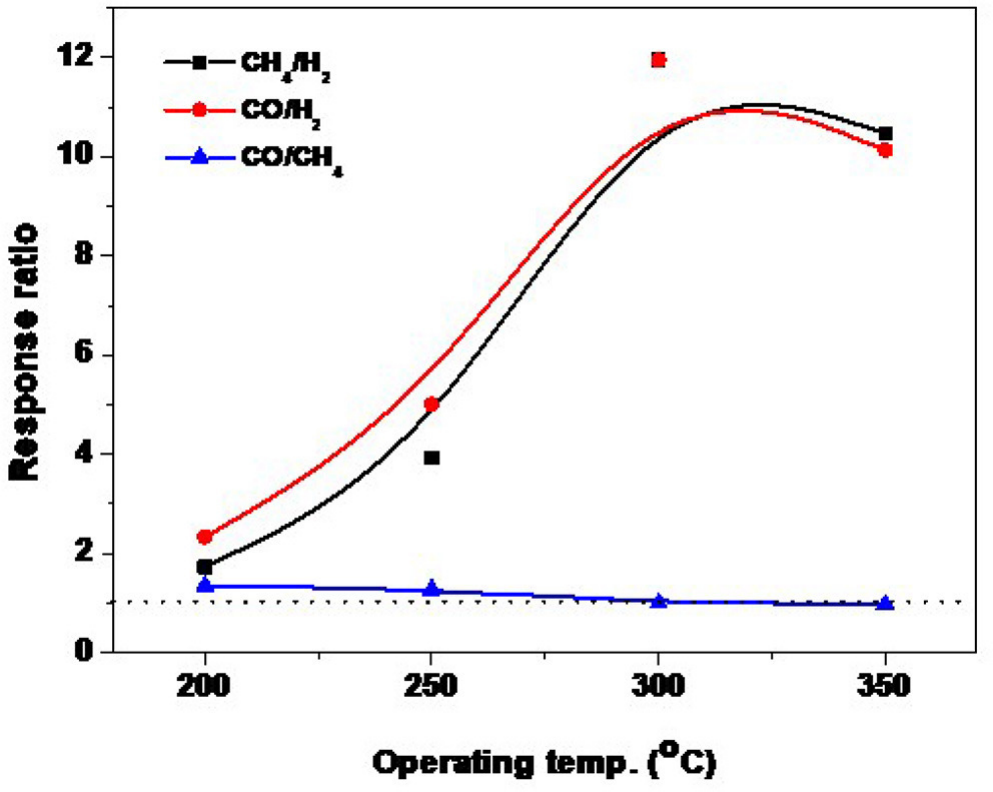
Temperature dependence of the response ratio for 1.0% CH_4_, 0.1% CO and 1.0% H_2_ for ZnO NRs.

Figure 9 shows the sensor evaluation toward the targeted gases at different operating temperatures. As mentioned above that the sensor of NRs is highly responsive toward the reducing gases, however, it is recommended for the high-temperature sensor. Figure 9 shows the evaluation curve of the response ratio of ZnO-NRs for CO and CH_4_ against H_2_, and CO against CH_4_ gas, respectively. The response ratio is expressed as the ratio of the gas response value to another gas (e.g., CO response value to H_2_ response value, α_CO/H2_ = S_CO_/S_H2_). The sensor showed a ratio higher than >1 for CO and CH_4_ against H_2_ gas at all temperature ranges. The maximum response ratio of ZnO-NRs for either CO or CH_4_ gas is ~11.7 at 300°C. For CO against CH_4,i_t is higher than >1.0 at 200, and 250°C, while it is ~1.0 at 300°C and <1.0 at 350°C. The sensor showed a high ratio for either CO or CH_4_ gases compared to H_2_ gas. It can be concluded that ZnO NRs is the optimum composition for the CO and CH_4_ gas sensor. The temperature dependence of the detection of both CO and CH_4_ gases by NRs is closely related to the surface activation at different sensing temperatures.

### Calibration Curve and Reversibility

**Figure 10:**
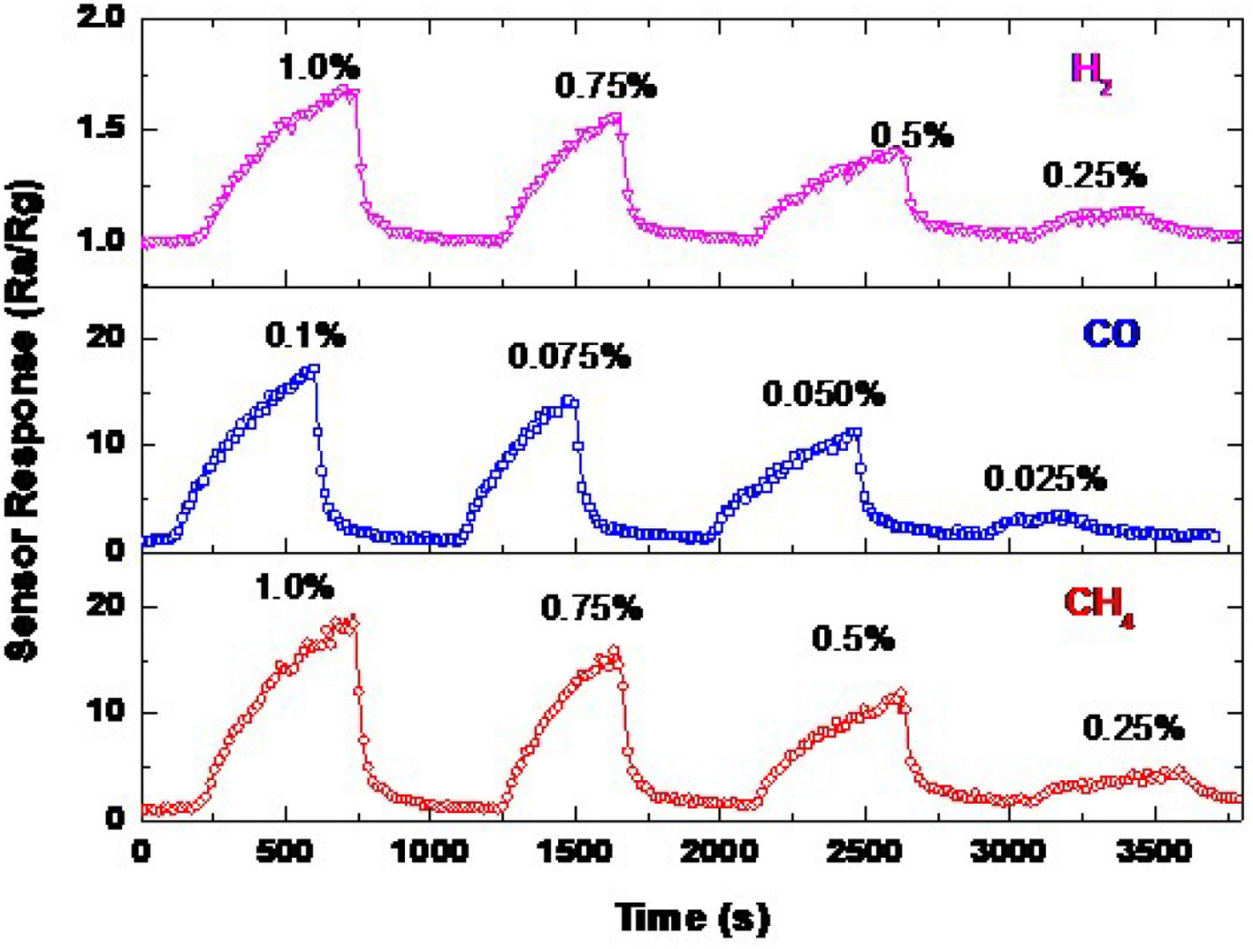
Sensor signals of ZnO NRs toward various concentrations of CH_4_, CO, and H_2_ at an operating temperature of 300°C.

The sensor signal of ZnO NRs for CH_4_, CO, and H_2_ measured a few months later at various concentrations at an operating temperature of 300°C is shown in Figure 10. The sensor response depends on gas concentration. The response to different gas concentrations was repeated, confirming the reliability of these sensors. It can be observed that the sensor is responding promptly to the change in the gas concentration. The sensor still responds to the lower concentrations of the gas even with fair sensitivity, especially for H_2_ gas, which exhibits the lower sensitivity compared to CH_4_ and CO gases. This measurement was carried out a few months later from the first measurement, thus, we may be able to state that the sensor signal is stable, and it is reversible. The sensor also provides almost an identical response value toward 1.0, 0.1, and 1.0% of CH_4_, CO, and H_2_, respectively, with a drift of <2.5%.

**Figure 11:**
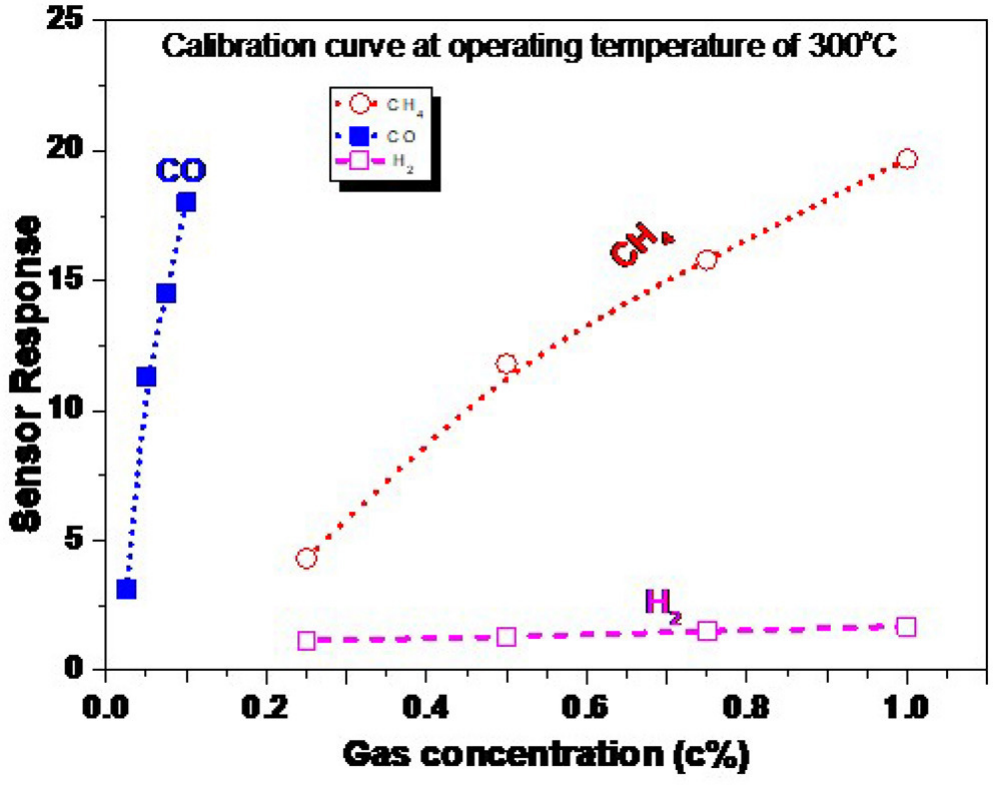
Calibration curve of ZnO NRs toward CH_4_, CO, and H_2_ at an operating temperature of 300°C.

The calibration curve of the ZnO NRs sensor measured at the most highly sensitive operating temperature of 300 °C is presented in Figure 11. This figure is extracted from Figure 10, where the gas concentration was changed from 1.0 down to 0.25% for CH_4_ and H_2_, and from 0.1 down to 0.025% for CO gas. The gas concentration was controlled by the adjustment of the flow rate, kept at 200 ml/min, between the synthetic air (21% O_2_, and 79% N_2_) and the air containing the maximal gas concentration of 1.0% CH_4_, 0.1% CO, and 1.0% H_2_. The gas concentration was kept much lower than the lower explosive level (LEL) for the targeted gases. The response is curve-like, where a soft increase in the sensor response is observed. There are two stages of the calibration curve of the present sensor, showing the non-linear behavior in general. The sensor responds respectably to the low and high concentration, which may be ascribed to the nature of the NRs shape, which allows the gases to diffuse deeply into the sensing layer to react with more oxygen species. To thoroughly address this behavior, a wide range of higher gas concentrations should be studied. These measurements may be carried out in the future, supported by theoretical bases.

## Conclusions

In summary, ZnO NRs have been successfully synthesized using a domestic microwave-assisted solution method and showed a smooth surface morphology and wurtzite hexagonal structure. The morphology and structure were studied by XRD, FESEM, and Raman spectroscopy. The sharp peak of Raman and XRD spectra exhibited a good crystallinity of the as-prepared ZnO NRs. When these NRs were applied for the gas sensing testing, they showed good performance toward the targeted gases such as CH_4_, CO, and H_2_. However, due to the smooth surface of the oxide NRs, the sensor was inactive at the low-temperature range of RT-150°C. Thus, it showed good sensitivity to all targeted gases especially CH_4_ and CO compared to H_2_, but at a higher temperature of 200–350°C. The advantage of the ZnO NRs sensing performance toward CO and CH_4_ compared to H_2_ gas was explained in terms of the possible gas sensing mechanisms. Where CO and CH_4_ gases can react with the oxide surface species of O^2−^ and O^−^ ions, in contrast with H_2_, which likely reacts with O^−^ or dissociates to H^+^. However, surface modification of ZnO nanostructures might be tuned to work at lower temperatures with good stability. Finally, we conclude that the fabricated ZnO NRs using the present method is very sensitive to CH_4_ and CO, where the sensitivity toward these two gases was very high compared to H_2_ gas. These smooth surface NRs can also be used as a high operating temperature sensor.

## Data Availability Statement

The raw data supporting the conclusions of this article will be made available by the authors, without undue reservation.

## Author Contributions

NS, FA, and CA: data curation and writing—original draft. AA, NS, and FA: formal analysis and methodology. AA: funding acquisition and supervision. AA and NS: writing—review and editing. All authors contributed to the article and approved the submitted version.

## Conflict of Interest

The authors declare that the research was conducted in the absence of any commercial or financial relationships that could be construed as a potential conflict of interest.
